# Size dependency of patch departure behavior: evidence from granivorous rodents

**DOI:** 10.1002/ecy.2800

**Published:** 2019-07-24

**Authors:** Francesco Cozzoli, Vojsava Gjoni, Alberto Basset

**Affiliations:** ^1^ Department of Biological and Environmental Sciences and Technologies University of the Salento S.P. Lecce‐Monteroni Lecce 73100 Italy

**Keywords:** foraging theory, giving up density, body size, predation, allometry, rodents

## Abstract

Individual size is a major determinant of mobile organisms’ ecology and behavior. This study aims to explore whether allometric scaling principles can provide an underlying framework for general patterns of resource patch use. To this end, we used giving‐up densities (GUDs), that is, the amount of resources remaining in a patch after a forager has quit feeding, as a comparative measure of the amount of resources exploited by a forager of any given size. We specifically tested the hypothesis that size‐dependent responses to both internal (energy requirement) and external (risk management) forces may have an effect on GUDs. We addressed this topic by conducting an extensive meta‐analysis of published data on granivorous rodents, including 292 GUD measurements reported in 25 papers. The data set includes data on 22 granivorous rodent species belonging to three taxonomic suborders (Castorimorpha, Myomorpha, and Sciuromorpha) and spans three habitat types (desert, grassland, and forest). The observations refer to both patches subject to predation risk and safe patches. Pooling all data, we observed positive allometric scaling of GUDs with average forager size (scaling exponent = 0.45), which explained 15% of overall variance in individual GUDs. Perceived predation risk during foraging led to an increase in GUDs independently of forager size and taxonomy and of habitat type, which explained an additional 12% of overall GUD variance. The size scaling exponent of GUDs is positive across habitat types and taxonomic suborders of rodents. Some variation was observed, however. The scaling coefficients in grassland and forest habitat types were significantly higher than in the desert habitat type. In addition, Sciuromorpha and Myomorpha exhibited a more pronounced size scaling of GUDs than Castorimorpha. This suggests that different adaptive behaviors may be used in different contexts and/or from different foragers. With body size being a fundamental ecological descriptor, research into size scaling of GUDs may help to place patch‐use observations in a broader allometric framework.

## Introduction

Body size is one of the most fundamental characteristics of an organism. It is linked to fundamental individual parameters such as metabolic, growth, and reproduction rates (Peters 1983, Calder 1984, West et al. 1997, Brown et al. 2004), as well as to physical aspects such as locomotion, dispersal, and space use (Bekoff and Mech 1984, Ritchie and Olff 1999, Jetz et al. 2004, Woodward et al. 2005). Larger organisms have higher total metabolic energy demand per unit of time (West et al. 1997, Kooijman 2000, Capellini et al. 2010) and must therefore maintain higher rates of resource harvesting and ingestion (Peters 1983, Hendriks 1999). However, harvesting and ingestion rates decrease as foragers harvest food from a patch as the unharvested resources become progressively more diluted and harder to find (Holling 1959a, b, Kotler and Brown 1990, Basset et al. 2012). To optimize foraging behavior, foragers are expected to exploit a patch if the relative ingestion rate of resources is higher than the average in the foraging area (i.e., marginal value theorem; Charnov 1976, Stephens and Krebs 1986) or if it balances the metabolic and fitness costs of foraging (Brown et al. 1988). Whenever resource availability limits the individual energy budget, larger foragers are expected to abandon the resource patches at higher resource density than smaller foragers (Basset 1995, Basset and DeAngelis 2007, Cozzoli et al. 2018). This is because total individual metabolic costs increase with size. Patches with low resource density may therefore represent valuable resources for small foragers while being unexploitable by large foragers (Holling 1992) because they do not allow the latter to maintain a sufficiently high ingestion rate (Basset et al. 2012). It is hypothesized that size‐dependent differences in foraging behavior may give rise to coexistence mechanisms with regard to a single resource on a multipatch scale (Wilson 1975, Basset 1995, Szabó and Meszéna 2006, Basset and DeAngelis 2007). They may also explain latitudinal patterns in the size distributions of herbivores such as the Bergmann's rule (Brown et al. 2017). This notion is supported by the observation that the lower individual total metabolic costs of smaller herbivores enable them to survive in areas where food plants are lower in abundance, and the lower metabolic cost per unit of mass and the greater digestive efficiency of large herbivores mean that they can survive on resources that are lower in quality (Belovsky 1997).

Differently sized foragers are also expected to experience different levels of predation risk (Stephens and Krebs 1986, Lima and Bednekoff 1999), because body size affects the likelihood of detection, attack, and capture by a predator and the costs of predator avoidance (Brooks and Dodson 1965, Urban 2007a, b, Thierry et al. 2011, Preisser and Orrock 2012). Although larger body sizes may confer protection, including size refuge (Urban 2007b) and influence the ability to react to attacks (Jackson and Dial 2011), larger foragers have a higher likelihood of being detected (Mech and Zollner 2002, Urban 2007a) and are also hunted by larger and more threatening predators (Brose et al. 2006, Tamburello et al. 2015). As a result of the complex interactions between body size and response to predation risk, reported cross‐taxon relationships are strongly dependent on prey and predator characteristics (Urban 2007b, Preisser and Orrock 2012). Environmental conditions may also have a complex role (Dial et al. 2008). As an example, heterogeneous and complex habitats such as forests and bushes may provide refuge from predation but also hiding places for lurking predators, and open habitats as deserts and bare lands increase the detectability of prey (Kelt et al. 2004).

The giving‐up density (GUD) framework (Brown et al. 1988, Brown and Kotler 2004, Bedoya‐Perez et al. 2013) provides a powerful experimental approach to investigate how differences in size scaling metabolic costs and perceived predation risks affect resource harvesting. Mobile animals’ foraging decisions can be quantified by measuring their GUD, i.e., the density of resources left when a forager decides to leave the resource patch. The GUD framework traditionally incorporates resource‐use determinants such as metabolic costs (Brown et al. 1994, Bozinovic and Vasquez 1999), predation risk (Arthur et al. 2004, Brown and Kotler 2004, Verdolin 2006, Kotler and Brown 2017), and missed opportunities (Olsson and Ngozi Molokwu 2007, Hagy et al. 2017). GUDs may vary relative to food densities in other accessible patches (marginal value theorem; Charnov 1976, Hagy et al. 2017) or across species and their habitats, depending on factors associated with perceived environmental quality (Brown 1989, Brown et al. 1994, Kelt et al. 2004, Wolf and Batzli 2004, Ceraldini and Chalfoun 2017). They also depend on the effort required to harvest the resource (Hughes et al. 1995, Abu Baker and Brown 2009) and are influenced by intra‐ (Berger‐Tal et al. 2015, Carthey and Banks 2015) and interspecific (Jones et al. 2001, Gutman and Dayan 2005) competition and by species‐specific preferences regarding resource quality (Brown and Morgan 1995, Garb et al. 2000, Horst and Venable 2018). Seasonal variations in GUDs have also been observed (Brown 1989, Meyer and Valone 1999, Ngozi Molokwu et al. 2008). Environmental temperature may have an effect on GUDs by adding additional thermoregulatory costs to foraging activities (Kotler et al. 1993, Bozinovic and Vasquez 1999, Falcy and Danielson 2013). Individual personality traits also play a significant role in determining GUDs. As an example, some individuals of Arnhem rock rats (*Zyzomys maini*; ;Cremona et al. 2015) and brushtail possum (*Trichosurus vulpecula*; Mella et al. 2015) are bolder than others in exploring high‐risk but high‐quality patches. The GUD determinants may interactively influence each other in complex ways (Kelt et al. 2004).

Individual forager size may have a wide‐ranging influence on GUDs because it affects the individual's resource requirements (Peters 1983, Kooijman 2000, Brown et al. 2004, Marquet et al. 2005), perception of resource patchiness and density (Holling 1992, Ritchie 1998, Haskell et al. 2002, Szabó and Meszéna 2006, Basset et al. 2012), and antipredator behavior (Urban 2007b, Thierry et al. 2011, Preisser and Orrock 2012). Few empirical studies (Bowers et al. 1993, Searle et al. 2005, Stenberg and Persson 2006, Cozzoli et al. 2018) have addressed size‐related trends in the GUDs of primary consumers. Higher GUDs with higher individual body masses have occasionally been observed in studies comparing different species (Brown et al. 1988, 1994, Kotler et al. 2002), but contrasting evidence has also been found (Smith and Brown 1991, Thorson et al. 1998).

In this study, we tested the hypotheses (1) that GUDs will scale as a positive allometric function of forager size (*M*), in accordance with GUD* = aM* ^*b*^, because of increasing total metabolic cost with increasing body size; (2) that GUDs scaling with the forager size in safe patches will differ from risky patches because of potential size dependency of predator avoidance costs, that is, that GUDs in risky patches will scale with a lower or higher scaling coefficient than in safe patches if, respectively, larger or smaller foragers have an advantage in managing predator avoidance costs; and (3) that observed trends will be consistent across a range of taxonomic groups and habitats. We addressed these topics by conducting an extensive meta‐analysis of published data on granivorous rodents, including 292 GUD treatments reported in 25 studies (Data S1, Metadata S1). The data set spans 22 granivorous rodent species belonging to three taxonomic suborders (Castorimorpha, Myomorpha, and Sciuromorpha). Observations were collected over three habitat types (desert, grassland, and forest). Seventy‐one percent of the GUD measurements were recorded in habitats with a high likelihood of predation (i.e., patches that are exposed or illuminated or in the presence of predators or a combination of two or three of these risk factors), and 29% were collected in safe conditions (i.e., sheltered and dark patches with no predator presence). The data set we used is one of the most complete (to our knowledge) on patch departure behavior ever assembled, taking into account size gradients across different species. Relating changes in patch departure behavior to forager size, as we did in this study, frames our observations within the context of ecological theories of allometric scaling (Brown et al. 2004) and size‐related species coexistence (Holling 1992, Szabó and Meszéna 2006, Basset and DeAngelis 2007).

## Materials and Methods

### Literature mining

We searched all papers indexed until 2017 in the SCOPUS^1^ and ISI Web of Knowledge^2^ research engines for the keywords “GUD” and “giving‐up density.” Within this group, we selected those studies focusing on granivorous rodents because of their high representation in the scientific literature and the high comparability of the methods applied. In all examined studies, the foraging trials involved seeds mixed in a matrix of loose sand, so that patches provide diminishing returns with resource depletion because of the dilution of the seeds in the sandy matrix. This means that the energy gain per unit of time decreases with the remaining amount of the resource, until reaching the level at which the forager decides to quit the resource patch (Kotler and Brown 1990, Morris 2001). In accordance with the availability of studies, we restricted the analyses to species belonging to the Castorimorpha, Myomorpha, and Sciuromorpha suborders and to observations collected in desert, grasslands, and forest habitats. We further selected studies focusing on comparison of the intraspecific effects of risks arising from (1) predator presence (i.e., predator decoys, scent, or live predators); (2) exposure of resource patches (i.e., absence of vegetation or other potential shelter from predators); and (3) patch illumination (i.e., full moon or artificial light), which makes the forager more detectable to predators. The experiments that gave rise to our data set were conducted in accordance with a factorial design including controls. This made it possible to codify the influencing factors of influence as binomial variables. Where the experimental design considered gradients of increasing disturbance (e.g., distance from cover, intensity of illumination, type of predator), we relied on the authors’ data analysis and interpretation to dichotomize the explanatory variable. Sources of risk that were not explicitly accounted for in the original experimental design were considered to be absent even if the studies did not explicitly state about the risk factors.

We discarded studies in which the observed GUD values were not clearly reported in the main text or figures and in which the foragers were not identified at the species level. To ensure internal comparability, we also discarded studies (1) in which the foragers were provided with resources other than the most commonly used (millet or sunflower seeds), (2) in which the seeds were dispersed in substrata other than the most commonly used (loose sand), (3) in which the seeds were dispersed in a disproportionately low volume of sand (<2 L) (4) in which the size of the food trays was not clearly reported; (5) in which repeated foraging episodes by multiple individuals of the same species were not allowed; (6) in which the foragers were provided with a disproportionately high amount of resources compared to other studies (>30 g). The first four conditions were imposed to reduce variability in GUDs arising from differences in food (Garb et al. 2000), substratum (Hughes et al. 1995), or resource dilution (Abu Baker and Brown 2009). Conditions 5 and 6 were imposed to avoid studies in which, because of low forager densities or restricted activity periods compared to the amount of exploitable resource, the observed quitting resource densities may be higher than the true GUDs (Price and Correll 2001). Our meta‐analysis finally included 25 studies (Metadata S1) covering a total of 22 rodents’ species (Table 1) and 292 experimental treatments (i.e., unique species–treatment combinations), for which the average GUD is reported. For each species and each experimental treatment within each paper, the average GUD per treatment was obtained from the main text or tables and, where not reported elsewhere, from figures (Data S1).

**Table 1:** List of the analyzed granivorous rodent species, ordered by habitat and suborder

Habitat	Suborder	Species	Size (g)	Observations	
				(*N*, total)	(*N*, risky)
Desert	Castorimorpha	*Perognathus amplus*	12	22	17
		*Chaetodipus penicillatus*	17.3	12	12
		*Chaetodipus baileyi*	37	16	14
		*Dipodomys merriami*	38	20	17
		*Dipodomys ordii*	52	12	10
		*Dipodomys deserti*	118	2	1
	Myomorpha	*Gerbillus allenby*	24	57	36
		*Gerbillurus tytonis*	28	6	4
		*Gerbillus pyramidium*	39	18	12
		*Acomys cahirinus*	45	22	16
		*Acomys russatus*	45	23	17
		*Jaculus jaculus*	55	2	1
		*Phyllotis darwini*	58	2	1
	Sciuromorpha	*Spermophilus tereticaudus*	125	14	7
		*Ammospermophilus harrisii*	126	14	7
Grassland	Myomorpha	*Sigmodon hispidus*	115	24	19
Forest	Sciuromorpha	*Tamias minimus*	47	2	1
		*Tamias striatus*	130	2	1
		*Spermophilus tridecemlineatus*	173	6	5
		*Tamiasciurus hudsonicus*	194	2	1
		*Spermophilus lateralis*	257	2	1
		*Sciurus niger*	800	12	8

Notes

Average species sizes were obtained from the ADW (Myers 2000) and AnAge (Tacutu et al. 2013) websites. Each specific observation is the average value of one experimental treatment within one study. Both the total number of GUD measurements and the number of measurements collected in risky conditions are reported at species level.

As a result of the heterogeneity of the investigated studies and of the conditions imposed to ensure internal comparability, the final data set design is skewed; 208 out of 292 treatments involved patches with a high likelihood of predation, and the remainder involved safe patches (Table 1). The majority of the species and observations pertain to the desert habitat type (15 species, 242 treatments) and to the Myomorpha (8 species, 154 treatments) and Castorimorpha (6 species, 84 treatments) suborders. GUDs are reported for all three suborders in the desert habitat type only (Myomorpha, 7 species, 130 treatments; Castorimorpha, 6 species, 84 treatments; Sciuromorpha, 2 species, 28 treatments). GUDs for 1 species only (*Sigmodon hispidus*, Myomorpha) are reported in the grassland habitat type (24 treatments), and GUDs for Sciuromorpha are only reported in the forest habitat type (6 species, 24 treatments) (Table 1).

Foraging species were identified by live trapping, camera observation, or footprint recognition. Estimates of average species size (g) were obtained from the ADW (Myers 2000)^3^ and AnAge (Tacutu et al. 2013)^4^ websites. The average weight of the analyzed species ranges from 12 g (*Perognathus amplus*) to 800 g (*Sciurus niger*). The Sciuromorpha suborder is characterized by a higher average species size (231 g [±238 SD]) than the Castorimorpha (46 g [±38 SD]) and Myomorpha (51 g [±28 SD]) suborders. As only Sciuromorpha species are reported for the forest habitat type, the specimens investigated in forest habitats are characterized by a higher average size (267 g [±271 SD]) than those from the desert (55 g [±37 SD]) habitat type. The only species present in the grassland habitat has an average weight of 115 g (Table 1).

Millet and sunflower seeds were used as the food resource. The amount of resource provided at the beginning and left at the end of the experiment was reported as the weight in grams or the number of seeds in the foraging patch; in the latter case, we converted the number of seeds left to grams according to the average seed weight. Millet seeds (228 treatments) were provided to small species (from 12 to 257 g), and sunflower seeds (64 treatments) were used across a larger size range (45–800 g; Data S1). Sunflower seeds are larger (ca. 50 mg/seed) and have a higher energy value per unit of weight (ca. 26 kJ/g) than millet seeds (ca. 14 mg/seed; ca. 14 kJ/g). For comparability therefore, we converted the seed weight to kJ in accordance with the seeds’ average energy values^5^ (Metadata S1). The results of the GUD analyses based on millet or sunflower seeds considered separately are available as an appendix (Appendix S1).

The trays used in the examined studies were rectangular or circular. Their surface area ranged from 480 to 2,700 cm^2^ in surface (50% of the treatments between 1,423 and 2,025 cm^2^) and the volumes of sand in which the seed were mixed ranged from 2 to 5 L (50% of the treatments between 2.5 and 4.25 L). Assuming that the sand matrix was evenly dispersed within the trays, the depth of the layer of sand in which the seed was buried varied from 0.8 to 4.2 cm (90% of the treatments between 1.5 and 3 cm; Data S1). Higher absolute GUDs per resource tray are expected to be observed when resources are diluted in a larger volume of sand or dispersed over a larger surface, so that the foragers need to spend more time and energy locating the seeds (Abu Baker and Brown 2009). To balance this effect, the amount of initial resource and GUD were standardized to the surface area of the food tray (kJ/m^2^). In theory, the standardization of GUDs by volume of sandy matrix would allow greater equalization of the effort required to extract the seeds, because it takes into account variations in the depth of the sandy matrix, as well as the surface area. However, we chose to standardize the GUD values per unit area because the latter is a more tractable parameter than volume and because the variation in depth of the sandy matrix is limited in the studies considered. For comparison, the results of the analyses of absolute GUD values independently of the size of the patch (kJ per tray), GUD values standardized to the volume of the sandy matrix (kJ/L), and GUD values standardized to the amount of initial resource (%) are available as an appendix (Appendix S1). In the examined studies, the initial resource density of the patches provided to the foragers varied from 78 to 5,416 kJ/m^2^ (50% of the treatments between 467 and 578 kJ/m^2^; Data S1).

The collected data set (which also includes 251 treatments excluded from the presented analyses) is available as Supporting Information to this paper (Data S1) and in the OSF repository (see Data Availability).

### Data analyses

The size scaling of GUDs was assessed by ordinary least‐squares linear regression. The species average body size (g) and GUDs (kJ/m^2^) were natural log transformed in order to express their relationship as a power law. Differences in GUD size scaling between risky and safe foraging conditions were assessed by linear analyses of covariance (ANCOVA) based on the pooled data. The relative importance of body size and risk level in explaining GUD variance were assessed by LMG metric (*R* ^2^ partitioned by averaging over orders; Lindeman et al. 1980).

A linear mixed model was used to test further for variations in GUDs in response to the fixed effect terms (size and cost of predator avoidance) and the random effect terms (habitat and suborder), fixed effects being expected to influence only the average of the response variable and random effects being expected to influence only the variance (Bolker et al. 2009, Zuur et al. 2009). We decided to consider habitat and suborder factors as random effect terms following Searle et al. (1992). Effects are fixed if they are interesting in themselves (in our case, the effect of individual size and cost of predator avoidance) or random if there is interest in the underlying population (in our case, unpredictable variation in GUDs resulting from differing habitat conditions or from differing specializations between suborders.). Unlike ordinary least‐squares regression, models with random effects do not have classic asymptotic theory for evaluating inference. Therefore, the significance of the random factors in generating variations in the intercepts and slopes was assessed by the likelihood ratio test (Giampaoli and Singer 2009). Models with varying degrees of complexity (allowing and not allowing interactions between fixed terms, and allowing variation across random terms either in intercept alone, or in both intercept and slope) were tested and evaluated by analysis of variance to select the minimal adequate model, that is, the model that best balances the likelihood of fit and the number of parameters estimated (Appendix S1: Tables S4, S5). The variables (and interactions between them) not considered in the minimal adequate model do not have significant influence on the response variable. The skewed character of the data set prevents consideration of the effect of interactions between habitat types and taxonomic suborders. Therefore, two different mixed models were fitted, one accounting for random variations between the type of habitat and the other accounting for random variation between taxonomic suborders. All analyses were performed within the R 3.3.2 free software environment (R Core Team, 2017) using the lmer (Bates et al. 2015), relaimpo (Grömping 2006), and sjPlot (Lüdecke 2018) packages.

## Results

### Preliminary data analyses

In the examined studies, the size of the foragers is weakly correlated with the surface area (Pearson's *r *=* *−0.29 [±0.1 95% CI]), volume (*r *=* *−0.21 [±0.11 95% CI]), and depth (*r *=* *0.24 [±0.11 95% CI]) of the sandy matrix in the experimental trays. The dimensions of the food trays are also correlated with the areal density of resource initially provided by the researchers (surface area: *r *=* *−0.47 [±0.09 95% CI]; volume: *r *=* *−0.41 [±0.09 95% CI]; depth: *r *=* *0.32 [±0.1 95% CI]) and with the measured GUDs (surface area: *r *=* *−0.45 [±0.1 95% CI]; volume: *r *=* *−0.44 [±0.09 95% CI]; depth: *r *=* *0.25 [±0.11 95% CI]). The areal density of resources originally provided is positively correlated with both the forager size (*r *=* *0.57 [±0.08 95% CI]) and the GUDs (*r *=* *0.80 [±0.04 95% CI]; Appendix S1: Fig. S1).

GUDs varied from a low of 17 kJ/m^2^ to a maximum of 2,546 kJ/m^2^ (50% of the observations between 104 and 414 kJ/m^2^; Data S1). Higher GUDs were measured in the forest (813 kJ/m^2^ [±566 SD]) and grassland (724 kJ/m^2^ [±333 SD]) than in the desert (227 kJ/m^2^ [±158 SD]) habitat types. On average, the GUDs of Myomorpha (286 kJ/m^2^ [±276 SD]) and Castorimorpha (302 kJ/m^2^ [±128 SD]) species were lower than those of Sciuromorpha species (442 kJ/m^2^ [±531 SD]; Data S1).

Preliminary data analyses also indicated that the various sources of risk (i.e., illumination, exposure, or predator presence), alone or in combination, do not significantly differ in terms of their effect on GUDs (Appendix S1: Fig. S2–S4, Table S1). Indeed, in general they involve a significant increase in GUDs compared to the patches where these sources of risk are all absent. Following these observations, GUD measurements were divided into two levels depending on whether they were collected in the presence of one or more explicit sources of risk introduced by the experimenters (risky), or in patches where these sources of experimental risk were all absent (safe). Across all habitats and suborders, GUDs were lower in safe patches (221 kJ/m^2^ [±338 SD]) than in risky ones (359 kJ/m^2^ [±297 SD]; Appendix S1: Fig. S4, Table S1).

### Size scaling of GUDs

**Figure 1:**
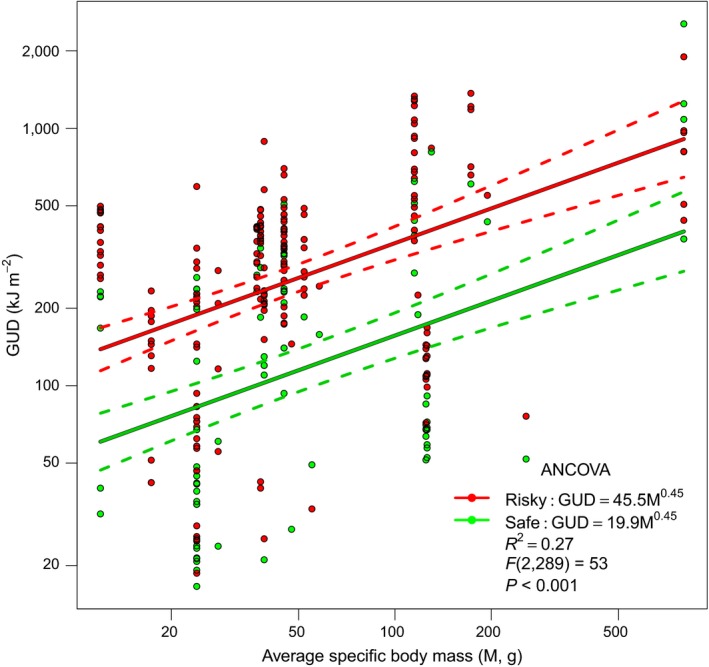
Average species body mass (M, g) scaling of giving up densities (GUD, kJ/m^2^) in risky (red) and safe (green) patches. The full lines represent the average scaling trend. The dashed lines represent the 95% confidence intervals around the average. The best selected model allows variations in intercept only across risk levels (Table 2).

Considering the pooled data, we observed a positive (exponent *b *=* *0.45 [±0.11 95% CI]) and significant (*F*_(2,289)_ = 53, *P *<* *0.001) scaling trend between GUDs and forager size. The estimated exponents under safe and risky conditions did not differ significantly, although risky foraging conditions did have a significant (*P *<* *0.001) effect on the intercept of the size scaling relationship (Fig. 1, Table 2). Size scaling was responsible for 15% of the explained variance in GUDs, and risk level explains 12% of the variance. The analyses of absolute GUD values independently of the size of the patch (kilojoules per tray, Appendix S1: Fig. S5, Table S2) or of GUD values standardized to the volume of the sandy matrix (kilojoules per liter, Appendix S1: Fig. S6, Table S2) gave similar results, whereas the GUD values standardized to the amount of initial resource (percent, Appendix S1: Fig. S7, Table S2) are independent from the forager size. We observed a positive size scaling of GUDs even when the analysis was restricted to experiments where sunflower seeds were the only food resource (Appendix S1: Fig. S8, Table S3), while we did not observe a positive size scaling of GUDs considering millet seeds only (Appendix S1: Fig. S9, Table S3).

**Table 2:** Comparison of linear models based on average species size (g), risk associated with foraging (safe vs. risky patches) and Giving Up Density of resources (GUD, kJ/m^2^). The continuous variables size and GUD were natural log transformed. The full‐interaction model and the cumulative model (best fit, bold) are shown

Predictors	log(GUD)~log(Size) × Species			**log(GUD)˜log(Size) + Species**		
	Estimates	95% CI	*P*	**Estimates**	**95% CI**	***P***
log(Intercept)	2.59	1.77–3.41	<0.001	**2.99**	**2.51–3.47**	<0.001
log (Size)	0.55	0.35–0.75	<0.001	**0.45**	**0.34–0.56**	<0.001
Risk	1.40	0.43–2.37	0.005	**0.83**	**0.60–1.05**	<0.001
log(Size):Risk	−0.15	−0.39–0.09	0.235			
Observations	292			**292**		
*R* ^2^/adjusted *R* ^2^	0.270/0.263			**0.267/0.262**		
AIC	767.1			**766.5**		

Variations in the relationship between size, risk level, and GUDs across different habitats and suborders were analyzed using linear mixed models and were found to be meaningful across habitat types and suborders (Table 3, Appendix S1: Tables S4, S5). The scaling of GUDs was always positive albeit minimal in the desert habitat type (*b *=* *0.09). In contrast, the forest habitat exhibited a pronounced size scaling trend (*b *=* *0.49). Only one species, which mirrors the overall trend, is present in the data set for the grassland habitat (Table 4). The scaling coefficients for Sciuromorpha and Myomorpha were higher (respectively, *b *=* *0.97 and *b *=* *1.39]) than Castorimorpha (*b *=* *0.21; Table 4). Even considering random variations between habitats and suborders, the effect of predation risk on GUDs was constant across all sizes, habitats, and suborders (Tables 3, 4, Appendix S1: Tables S4, S5).

**Table 3:** Summary of the most adequate linear mixed models based on average species size (g), risk associated with foraging (safe vs. risky patches) and Giving Up Density of resources (GUD, kJ/m^2^), accounting for random variation across habitat types (left) and suborders (right). The continuous variables size and GUD were natural log transformed

Predictors	Habitat			Suborder		
	Estimates	95% CI	*P*	Estimates	95% CI	*P*
log(Intercept)	3.15	1.86–4.44	0.296	1.31	−1.81–4.43	0.496
log(Size)	0.45	0.04–0.86	0.322	0.85	0.15–1.56	0.141
Risk	0.75	0.54–0.97	**<0.001**	0.59	0.40–0.79	**<0.001**
Random effects						
σ^2^	0.70			0.53		
τ_00_	1.10 _Habitat_			7.33 _Suborder_		
τ_11_	0.12 _Habitat.log(Size)_			0.37 _Suborder.log(Size)_		
ρ_01_	−1.00 _Habitat_			−0.96 _Suborder_		
ICC	0.61 _Habitat_			0.93 _Suborder_		

**Table 4:** Estimates produced by the linear mixed model in Table 3

Factor	Level	log(Intercept)	95% CI	log(Size)	95% CI	Risk	95% CI
Habitat	Desert	4.23	3.21–7.4	0.09	−0.96–0.42	0.75	0.54–0.97
	Forest	2.19	0.10–7.76	0.77	−0.23–1.29	0.75	0.54–0.97
	Grassland	3.03	−2.51–5.02	0.48	0.18–2.00	0.75	0.54–0.97
Suborder	Castorimorpha	4.38	2.10–12.79	0.2	−1.65–0.75	0.59	0.40–0.79
	Myomorpha	−0.39	−7.38–3.22	1.39	0.73–3.12	0.59	0.40–0.79
	Sciuromorpha	−0.06	−6.77–3.90	0.97	−0.12–2.29	0.59	0.40–0.79

Habitat and suborder are considered to be sources of random variance. 95% confidence intervals around estimates have been estimated via 9,999 parametric bootstrap iterations.

## Discussion

In line with our hypotheses, we observed positive scaling of GUDs with average species body size. We also observed a consistent increase in GUDs related to the costs of predator avoidance, which was maintained across habitat types and taxonomic suborders of rodents. This increase is proportional to the forager size and independent of the forager taxonomy, as well as of habitat type. The positive scaling of GUDs with size was maintained across different habitats and suborders, albeit near zero in the desert habitat and weak for the Castorimorpha suborder. Considering that our observations are the product of a meta‐analysis based on separate experiments that were not originally designed to test for the effect of the foragers’ size variations on GUDs, we will consider the possible limitations before discussing the initial hypotheses in the light our findings.

### Limitations

The positive correlation between forager size and the amount of resources provided by the researchers is an experimental artefact that serves to avoid giving larger foragers patches with resource levels that are already below their GUD and to avoid giving smaller foragers an amount of resources in excess of what they can process during the observational time. Although it could be argued that this may create a bias in our analyses, it must be considered that for a given type of forager, resource patches that have a similar total foraging cost but differed in initial resource density tend to result in similar GUDs (Kohlmann and Risenhoover 1998, Morris 2001, Vásquez et al. 2006, Abu Baker and Brown 2009) and so the proportion of resources harvested generally increases with initial resource density independently from the size of the foragers (Appendix S1: Fig. S7, Table S2). Any failure to equalize GUDs across similar treatments seems to result from experimental conditions that we excluded from our analyses, such as imperfect patch assessment (Olsson et al. 1999), low forager densities, or restricted activity periods (Price and Correll 2001).

In order to obtain a sufficient number of observations and a broad size range, the analysis took account of GUDs observed for two types of seeds (sunflower and millet) that differed in terms of size and energy content. Although we converted the seeds’ weight into kilojoules for comparability, differences between the two seed types could still generate a bias because larger seeds are more likely to be detected, resulting in lower GUDs than smaller seeds (Garb et al. 2000). However, in the examined studies, the larger sunflower seeds were provided mostly to the larger foragers. Thus, the higher detectability of the larger seeds should have led to lower GUDs for the larger foragers, so the differences in seed size are not likely to have generated a false positive in our main finding, that is, that GUDs scale positively with size. Furthermore, we observed a positive size scaling of GUDs even when the analysis was restricted to experiments where sunflower seeds were the only food resource (Appendix S1: Fig. S8, Table S3). Considering millet seeds only, we did not observe a positive size scaling of GUDs, possibly because of the narrower size range for which these data are available (Appendix S1: Fig. S9, Table S3).

It should also be noted that our analyses do not account for the effects of population density on GUDs (which the analyzed literature considers only in very few cases). As population density increases, so does the overall consumer pressure on resources, resulting in a lower per‐capita GUD as the population's overall requirements increase (Davidson and Morris 2001, Ovadia and Zu 2003, Berger‐Tal et al. 2015, Carthey and Banks 2015, Cozzoli et al. 2018, Menezes et al. 2019). Although our analyses did not take account of studies reporting low forager densities or restricted activity periods compared to the amount of exploitable resource, changes in GUDs arising from variations in population densities may still have contributed to the unexplained variance in our analyses.

Finally, potential bias in the analyses may arise from the fact that the data set was not designed to independently test for the effects of habitat and taxonomic variation on the size scaling of GUDs. For example, the largest suborder, Sciuromorpha, is the only one for which observations were available in the forest habitat type and only one species was considered for the grassland habitat type. It is thus possible that interactions between phylogenetic and habitat aspects may have skewed our estimates.

### Hypothesis 1: Size scaling of GUDs

The observed positive size scaling of GUDs is in agreement with both theoretical expectations (Ritchie 1998, Ritchie and Olff 1999, Haskell et al. 2002, Basset and DeAngelis 2007, Brown et al. 2017) and previous empirical evidence (Bowers et al. 1993, Brown et al. 1994, Searle et al. 2005, Stenberg and Persson 2006, Cozzoli et al. 2018). Positive size scaling of GUDs can be interpreted as an effect of the positive size scaling of the individual metabolic cost of foraging. Larger foragers, having higher overall metabolic costs compared to smaller foragers, reach earlier the threshold level of energy gain rate that requires the abandonment of the patch. Consequently, they leave the resource patch at higher amounts of resources (Basset 1995). Larger foragers may eventually compensate for this disadvantage with their greater mobility (Mittelbach 1981, Biewener 1989) and hence higher probability of finding new and more profitable resource patches (Brown et al. 1994, Basset and DeAngelis 2007).

The size scaling exponent of GUDs we observed (0.45 [±0.11 95% CI]) is significantly lower than the 0.75 or 0.66 expected for rodents on the basis of the size scaling of individual energy requirements alone (Capellini et al. 2010), indicating that larger foragers, despite having a higher absolute GUD than smaller ones, are able to use more resources in proportion to their energy requirements. This observation is consistent with the expectation that handling ability scales more favorably (i.e., with a higher scaling coefficient) with body size than metabolic foraging costs (Brown et al. 1994, 2017). Larger foragers may indeed be able to maintain a relatively higher harvest rate by improving their collection behavior (Dukas and Kamil 2001, Catania and Remple 2005, Mella et al. 2018) and by learning (Ishii and Shimada 2010). Furthermore, the costs of thermoregulation per unit of mass of rodents decrease as size increases (Bozinovic and Rosenmann 1989), so that larger individuals are advantaged when foraging in extreme temperatures. However, by means of these mechanisms, larger foragers can only partially compensate for their higher basal metabolic cost of foraging, resulting in a size scaling of GUDs that is still positive but characterized by a lower scaling coefficient than would be expected on the basis of total metabolic costs alone (Brown et al. 2017). This may allow larger foragers to reduce the size gap necessary for coexistence on the same resource with smaller competitors (Basset and DeAngelis 2007), and it is a necessary condition for explaining the increase in average size with latitude (i.e., Bergmann's rule) on the basis of foraging allometries (Brown et al. 2017).

### Hypothesis 2: Effect of predation risk on the size scaling of GUD

In addition to killing, predators also have nonlethal effects on prey because they impose additional fitness costs on foraging activity (Hughes et al. 1994, Bouskila 1995, Arthur et al. 2004, Brown and Kotler 2004, Kelt et al. 2004, Kotler et al. 2004, Verdolin 2006). Consistent with this consideration, GUDs doubled in the presence of predation risk across the whole size gradient included in our meta‐analysis. It is noticeable that the amount of resource left in the patch in response to predation risk increases with forager size *in proportion to* the increase observed in safe patches, instead of increasing by a fixed quantity. This supports the hypothesis that foragers evaluate resources on the basis of their energy requirements rather than as an absolute amount (Basset et al. 2012).

Our analyses show that although the cost of predator avoidance is the main determinant of GUD, with a greater weight than metabolic costs for foragers of a comparable size (Brown 1989, 1999, Brown et al. 1994), it actually explains a similar portion of GUDs variance to size scaling across the analyzed size range of 788 g. In other words, smaller foragers in risky conditions may have a similar GUDs to larger foragers in safe conditions. This could indicate that coexistence mechanisms related to spatial and temporal heterogeneity (Belovsky et al. 1989) and to the species specificity of predation risk intensity (Brown 1989, Lima and Bednekoff 1999, Brown and Kotler 2004, Verdolin 2006) may act in addition to fundamental resource portioning rules based on size scaling of individual energy requirements and ingestion rates (Basset 1995, Basset and DeAngelis 2007).

### Hypothesis 3: Consistency of the size dependency of GUDs across habitats and taxa

GUDs in the forest and (as far as can be assessed) grassland habitat types scale positively with size, while the relationship is weak in the desert habitat type. This suggests that physiological and behavioral adaptation to the resource scarcity and harsh climatic conditions typical of deserts (Willmer 2009) may be stronger than size scaling of individual energetics in determining patch departure behavior and may allow larger individuals to exploit relatively small resource patches. Animals in poorer environments are indeed expected to have lower GUDs than animals in richer environments (Persson and Stenberg 2006). Moreover, although body mass is the main determinant of energy requirements in small mammal species, climatic and biogeographical factors may also exert significant influence (Lovegrove 2013). Species belonging to dry habitat types have lower and less predictable basal metabolic rates than species of the same weight belonging to mesic habitat types (Rezende et al. 2004, Lovegrove 2013), possibly as an adaptation to the scarcity of resources and water (Willmer 2009). Finally, the desert habitat type is the most represented in our data set in terms of taxonomic diversity. Therefore, it is possible that differences in foraging strategies between taxa may mask the size scaling of GUDs in this habitat. Consistent with this consideration, we also detected significant variation in the scaling exponent of the GUD ~ size allometric relationship across different suborders of rodents. Although Myomorpha and Sciuromorpha exhibit steep positive scaling of GUDs with size, Castorimorpha GUDs are less dependent on size. This indicates that large Castorimorpha may adopt a different foraging strategy, characterized by a higher ability to exploit resources at the single‐patch scale. Indeed, the three larger species of Castorimorpha considered in our analysis belong to the genus *Dipodomys* and are known to be competitively dominant through aggressive defense of territory, fast rate of resource harvesting and wide range of microhabitat use, including open microhabitats that are generally avoided by smaller species (Price et al. 2000). *Dipodomys* also have cheek pouches that can increase their harvest rate and storing capacity of seeds (Emerson et al. 2018). These features could represent some adaptations of this genus to achieve relatively low GUD and sustain relatively large body in a resource‐poor habitat like the desert.

Our meta‐analysis shows that the predation‐driven increase in GUDs is independent of forager taxonomy, as well as of habitat type. A possible interpretation of this trend is that predation pressure is evenly distributed along the size gradient because the existence of species‐specific and habitat‐specific predators smooths out the effects of size, taxonomy, and habitat type (Preisser and Orrock 2012).

## Conclusion

Although broad allometric patterns have been explored in fields such as energetics, biogeography, community ecology, and evolutionary ecology, research into the effect of body‐size scaling on foraging behavior has lagged behind (Dial et al. 2008). Traditionally, it is assumed that short‐term foraging decisions are driven by extrinsic variation in environmental quality (Persson and Stenberg 2006) and are strongly influenced by the level of risk experienced during foraging (Verdolin 2006, Kotler and Brown 2017). The results of this study show that the intrinsic effect of size should also be regarded as a major determinant of foraging decisions. Our observations thus highlight the role of spatial patchiness of resources in determining the outcome of competitive interactions (Szabó and Meszéna 2006). For a large forager, the same amount of resources will have a lower perceived value if they are distributed in many small patches rather than in few large ones (Basset et al. 2012). Interaction between metabolic requirements and locally perceived resource availability may account for the observed power law relationship between forager population density and body size. By quantifying the positive size scaling of GUDs, this study offers a measure of perceived resource availability that is more realistic than overall biomass or resource density (Basset et al. 2012). The patterns we describe in this work may help to parameterize theoretical models of energy carrying capacity (van Gils et al. 2004, Hagy and Kaminski 2015) and size‐related species coexistence (Szabó and Meszéna 2006, Basset and DeAngelis 2007). The notion of size operating as a possible ecological constraint on primary consumer foraging behavior may represent an important direction for future research into space use and environmental carrying capacity for primary consumers. As an example, size‐related constraints on patchy resource use may help to explain the steep rate at which home range increases with primary consumer size (Reiss 1988, Basset and Ponti 1992, Tamburello et al. 2015, Ofstad et al. 2016). The size scaling of GUDs may have implications for management and conservation purposes, for example, by helping to determine the spatial requirements of target species, or to manipulate resource distribution so as to favor foragers of a certain size. As the scaling of metabolic rates with individual size is a fundamental driver of community and trophic interactions (Brown et al. 2004), it may eventually be possible to include other GUD determinants such as forager population density or missed opportunity costs in a common framework of size‐based models.

## Data Availability

Data are available from the Open Science Framework at https://doi.org/10.17605/osf.io/xqhn9

## Footnotes

1
https://www.scopus.com

2
https://webofknowledge.com

3
http://animaldiversity.org/accounts/Rodentia/

4
http://genomics.senescence.info

5
http://www.foodnutritiontable.com
