# Supplementary material for: Size dependency of patch departure behavior: evidence from granivorous rodents
Source: Ecology. 2019 Jul 24;100(10):e02800. doi: 10.1002/ecy.2800 (PMC6852180; doi:10.1002/ecy.2800)
Supplement: Supplementary file 1 [file ECY-100-na-s001.pdf]

Supporting Information. Francesco Cozzoli, Vojsava Gjoni, and Alberto Basset. 2019.

Size dependency of patch departure behavior: evidence from granivorous rodents. *Ecology*.

Appendix S1

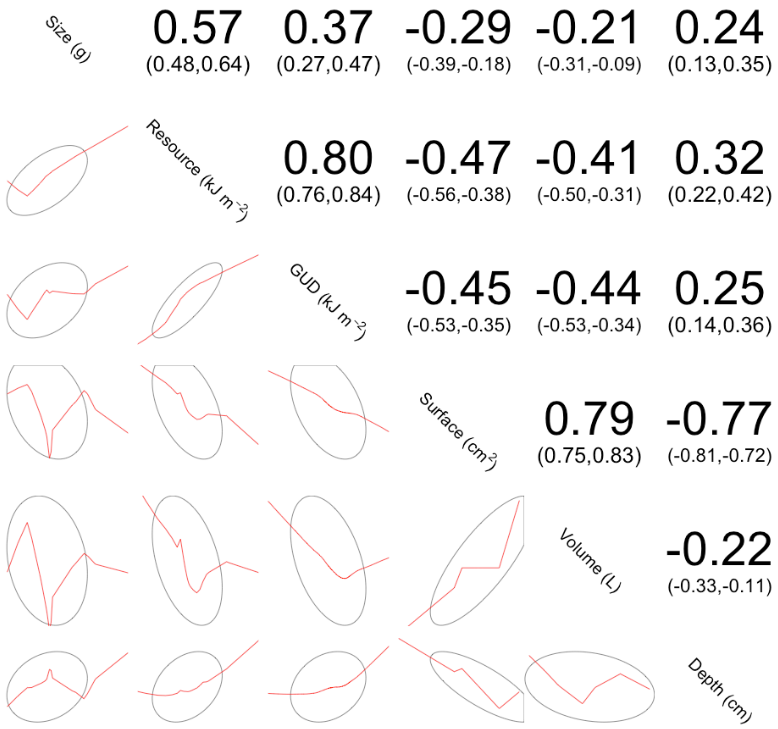

Figure S1: Correlation among average species size of rodents (g), amount of resource initially provided (kJ m^{-2}), Giving Up Density (kJ m^{-2}) and dimensions of the food trays (surface, cm^{-2}; volume, L; depth, cm). The upper panel show the Pearson correlation coefficient [± 95% CI] between couple of measures (diagonal). The lower panel show the average trend (red line) and the data dispersion (95% of the observations within the black ellipse).

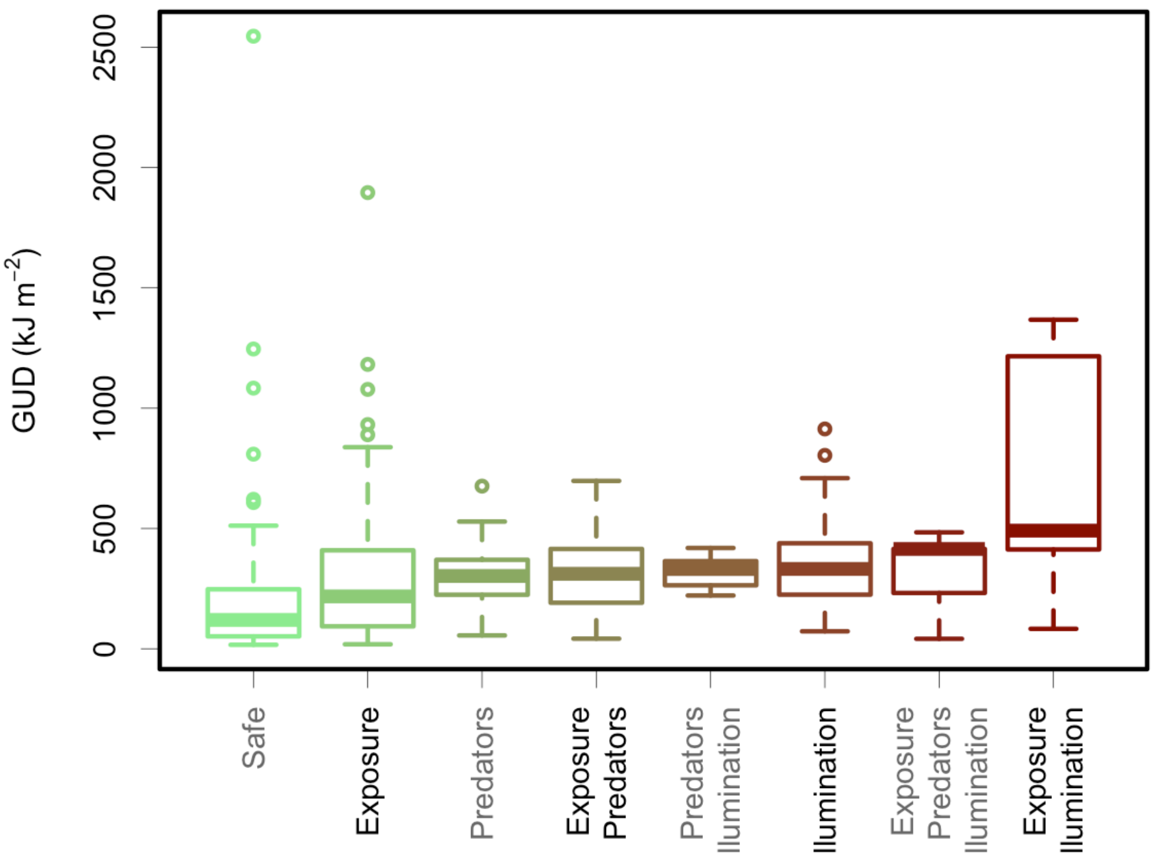

Figure S2: Investigated rodents' Giving Up Densities (GUD, kJ m^{-2}) with respect to 3 different types of risks (Exposure, Illumination, Predation) alone or in combination (Table S1). The lower and upper box boundaries indicate the 25th and 75th percentiles, respectively; the line inside box indicates the median; the lower and upper error lines indicate the 1.5 standard deviations interval around the average; circles indicate data falling outside the latter interval (outliers).

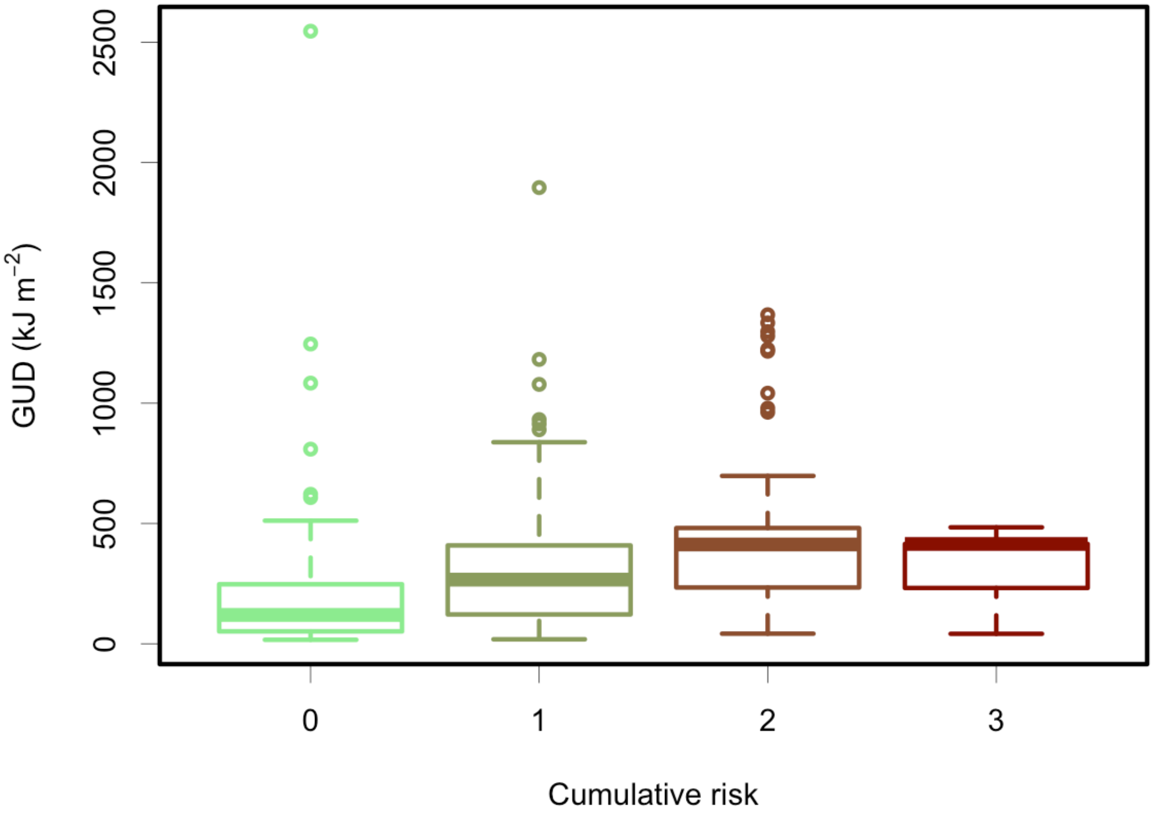

Figure S3: Investigated rodents' Giving Up Densities (GUD, kJ m^{-2}) with respect to the number of risks sources (Exposure, Illumination, Predation) used in the treatment (Table S1). The lower and upper box boundaries indicate the 25th and 75th percentiles, respectively; the line inside box indicates the median; the lower and upper error lines indicate the 1.5 standard deviations interval around the average; circles indicate data falling outside the latter interval (outliers).

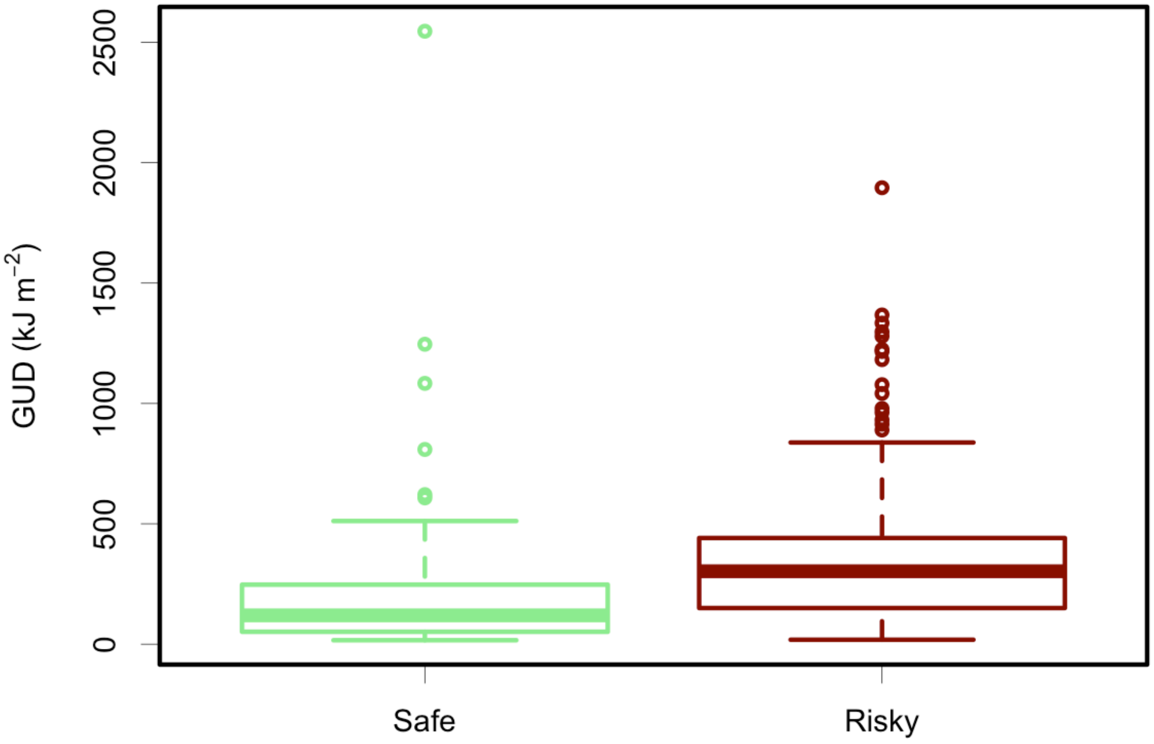

Figure S4: Investigated rodents' Giving Up Densities (GUD, kJ m^{-2}) with respect to the number of risks sources (Exposure, Illumination, Predation) used in the treatment (Table S1). The lower and upper box boundaries indicate the 25th and 75th percentiles, respectively; the line inside box indicates the median; the lower and upper error lines indicate the 1.5 standard deviations interval around the average; circles indicate data falling outside the latter interval (outliers).

Table S1: ANOVA model of the variations of GUD (kJ m^{-2}) respect to 3 different types of risks (Exposure, Illumination, Predation) alone or in combination (left column), to the cumulative number of risks sources (central column), and to the presence/absence of risk (right column)

	**Different combinations†**			**Cumulative‡**			**Binomial§**		
Predictors	*Estimates*	*95% CI*	*p*	*Estimates*	*95% CI*	*p*	*Estimates*	*95% CI*	*p*
Intercept	221.26	158.34 – 284.19	<0.001	221.26	156.23 – 286.30	<0.001	221.26	155.08 – 287.44	<0.001
Exposure	82.87	-2.11 – 167.84	0.057						
Predators	99.59	-41.12 – 240.30	0.166						
Exposure Predators	96.99	-46.50 – 240.49	0.186						
Predators Illumination	98.71	-114.69 – 312.10	0.365						
Illumination	144.07	12.67 – 275.46	0.032						
Exposure Predators Illumination	118.68	-66.25 – 303.61	0.209						
Exposure Illumination	502.54	361.83 – 643.25	<0.001						
Risk_Lev1				95.58	14.15 – 177.00	0.022			
Risk Lev2				271.08	163.93 – 378.22	<0.001			
Risk_Lev3				118.68	-72.44 – 309.80	0.225			
Risk							138.14	59.73 – 216.55	0.001

Note: Total observations are 292.

† R^2 / adjusted R^2 = 0.150 / 0.129
‡ R^2 / adjusted R^2 = 0.079 / 0.069
§ R^2 / adjusted R^2 = 0.039 / 0.036

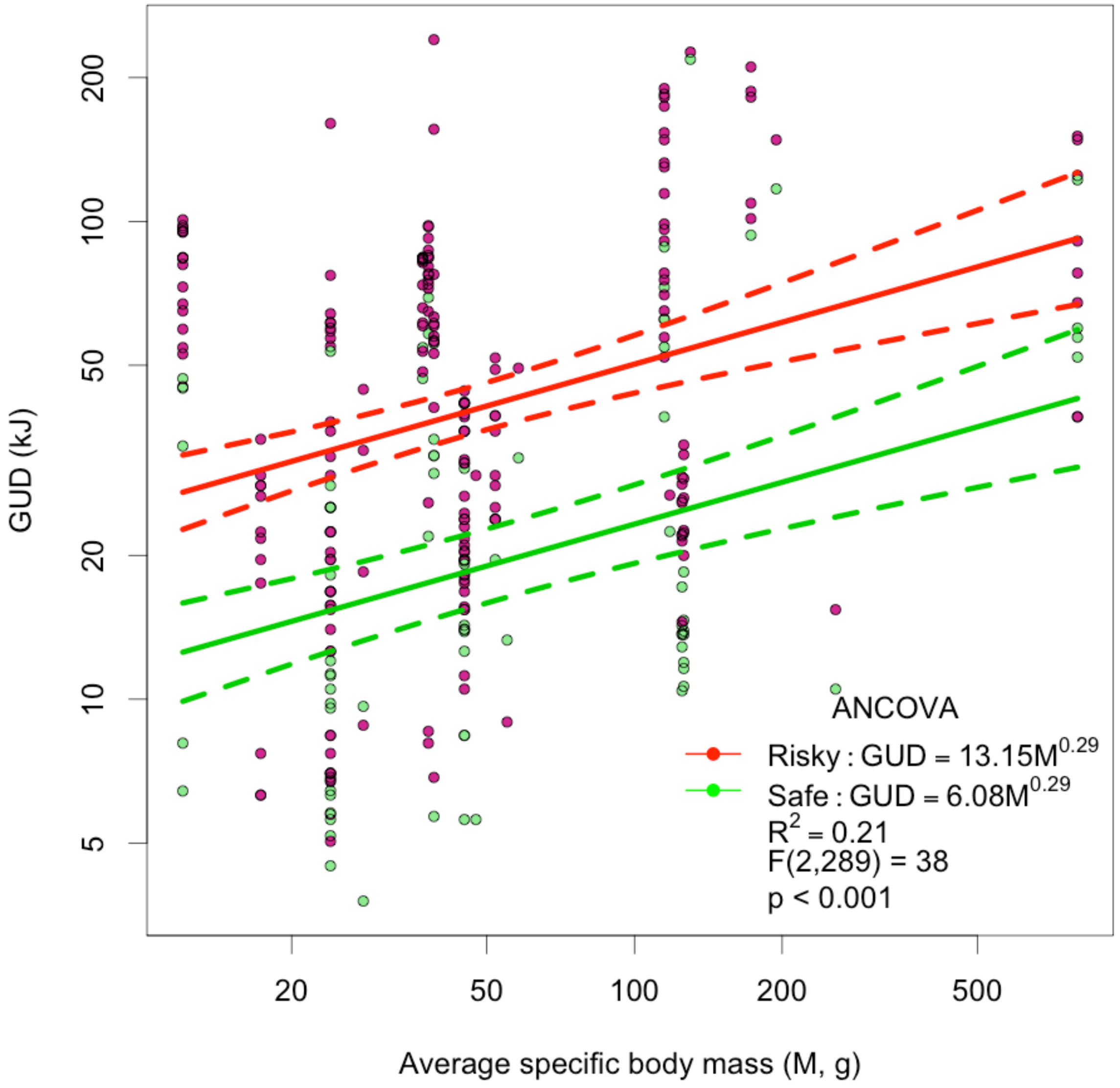

Figure S5: Average species body mass (M, g) scaling of Giving Up Densities (GUD, kJ per food tray) in risky (red) and safe (green) patches. The full lines represent the average scaling trend. The dashed lines represent the 95% Confidence Intervals around the average. The best selected model allows variations in intercept only across risk levels (Table S2).

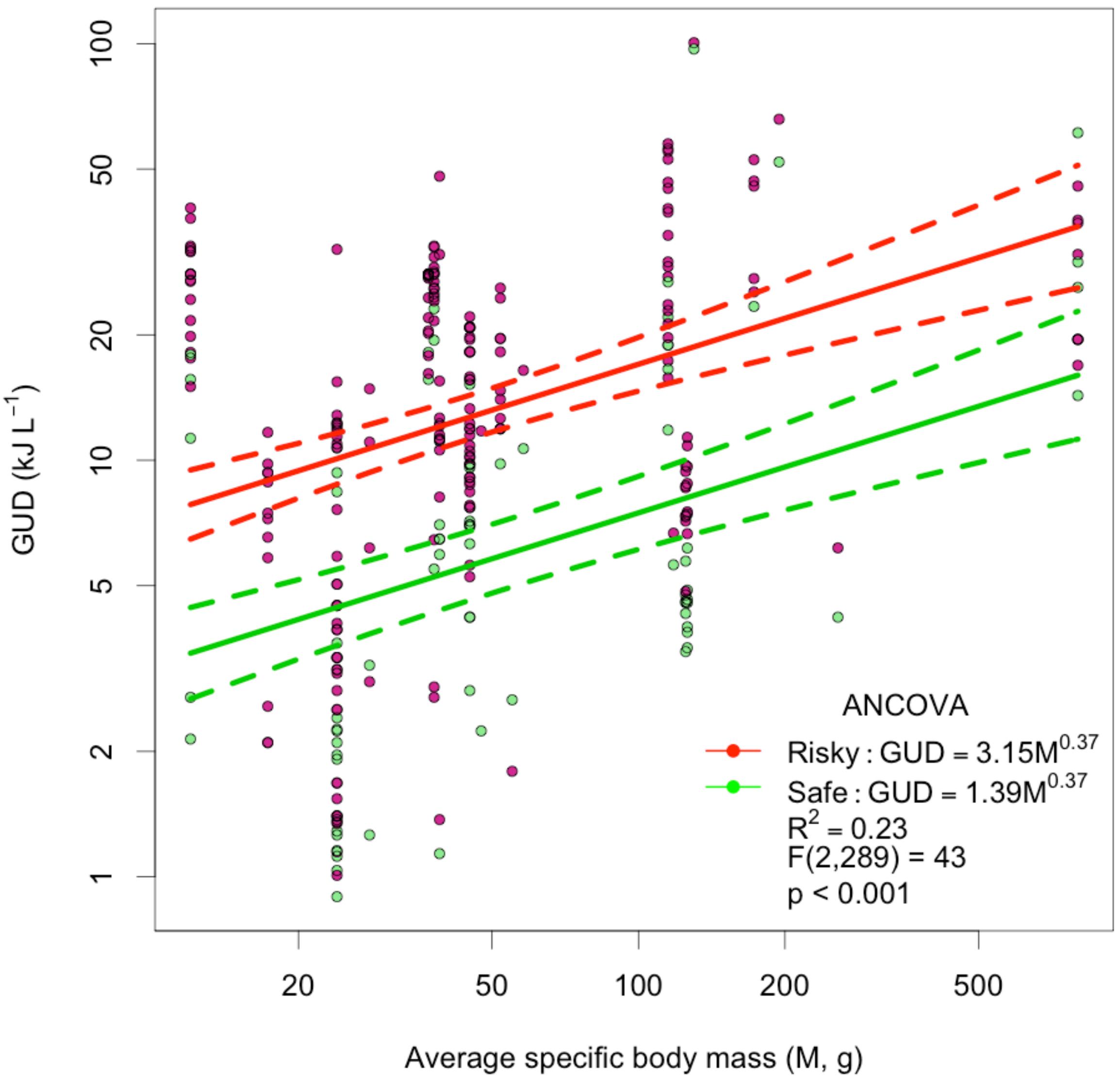

Figure S6: Average species body mass (M, g) scaling of Giving Up Densities (GUD, kJ L^{-1}) in risky (red) and safe (green) patches. The full lines represent the average scaling trend. The dashed lines represent the 95% Confidence Intervals around the average. The best selected model allows variations in intercept only across risk levels (Table S2).

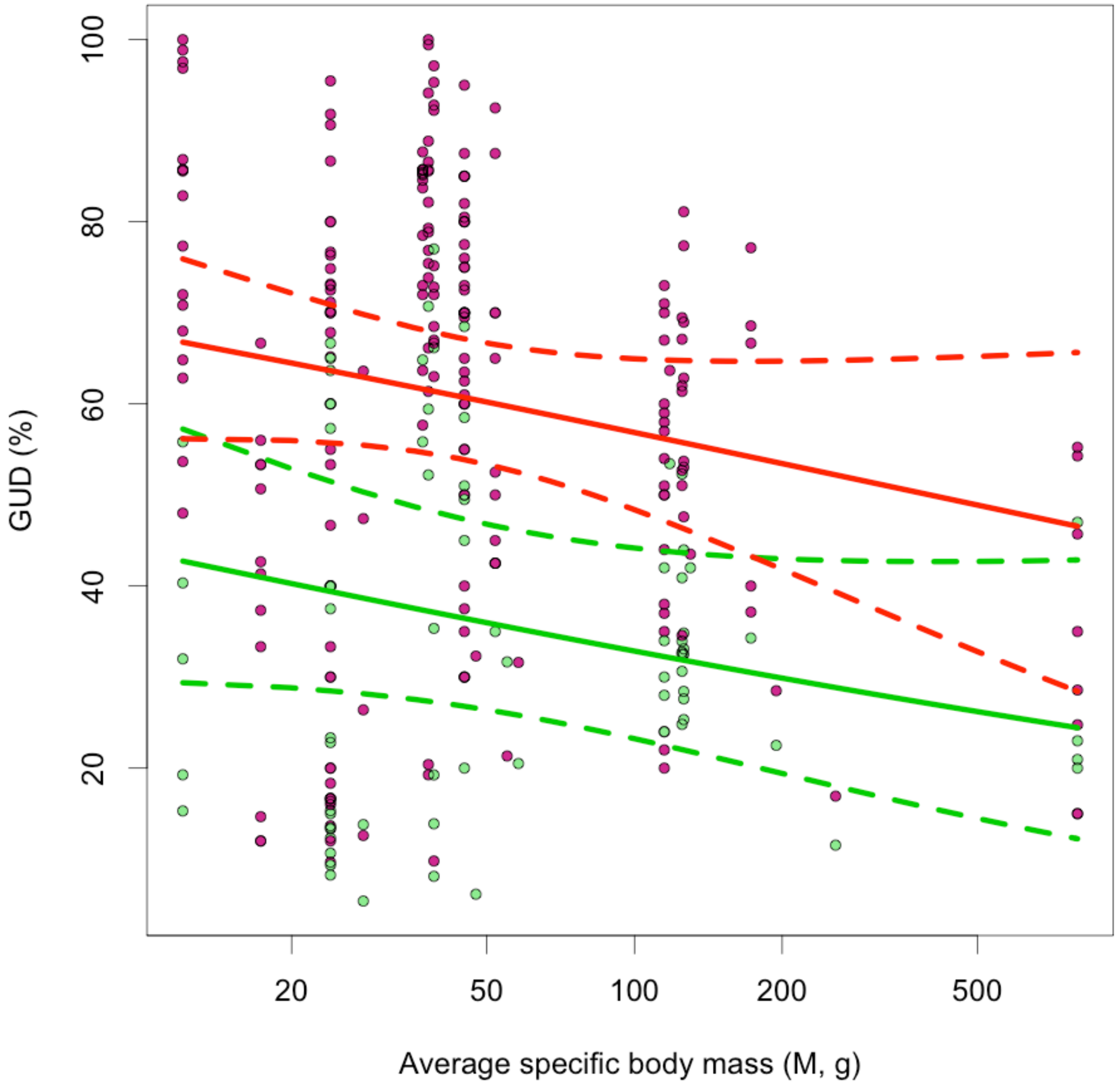

Figure S7: Average species body mass (M, g) scaling of Giving Up Densities (GUD, % of the resource initially provided) in risky (red) and safe (green) patches. The full lines represent the average scaling trend. The dashed lines represent the 95% Confidence Intervals around the average. The best selected logistic model does not detect significant size scaling (Table S2).

Table S2: Comparison of linear models based on average species size (g), risk associated with foraging (safe vs. risky patches) and different measures of Giving Up Density of resources; kJ per food tray (kJ, left), kJ per litre of sandy matrix (kJ L^{-1}, central) or percentage of resource left at the end of foraging respect to the provided amount (%, right). The continuous variables were natural log transformed or, in the case of percentage left, logit transformed. The full-interaction model and the cumulative model (best fit, bold) are shown.

	**GUD (kJ per food tray)†**			**GUD (kJ L^{-1})‡**			**GUD (%)§**		
Predictors	*Estimates*	*95% CI*	*p*	*Estimates*	*95% CI*	*p*	*Odds Ratios*	*95% CI*	*p*
log(Intercept)	1.81	1.36 – 2.25	<0.001	0.33	-0.15 – 0.81	0.181	1.22	0.40 – 3.72	0.722
log(Size)	0.29	0.19 – 0.39	<0.001	0.37	0.26 – 0.48	<0.001	0.82	0.63 – 1.06	0.132
Risk	0.77	0.56 – 0.98	<0.001	0.82	0.60 – 1.05	<0.001	2.69	1.59 – 4.57	<0.001

Note: Total observations are 292.

† R^2 / adjusted R^2 = 0.208 / 0.202
‡ R^2 / adjusted R^2 = 0.231 / 0.225
§ R^2 / adjusted R^2 = 0.057 / 0.238

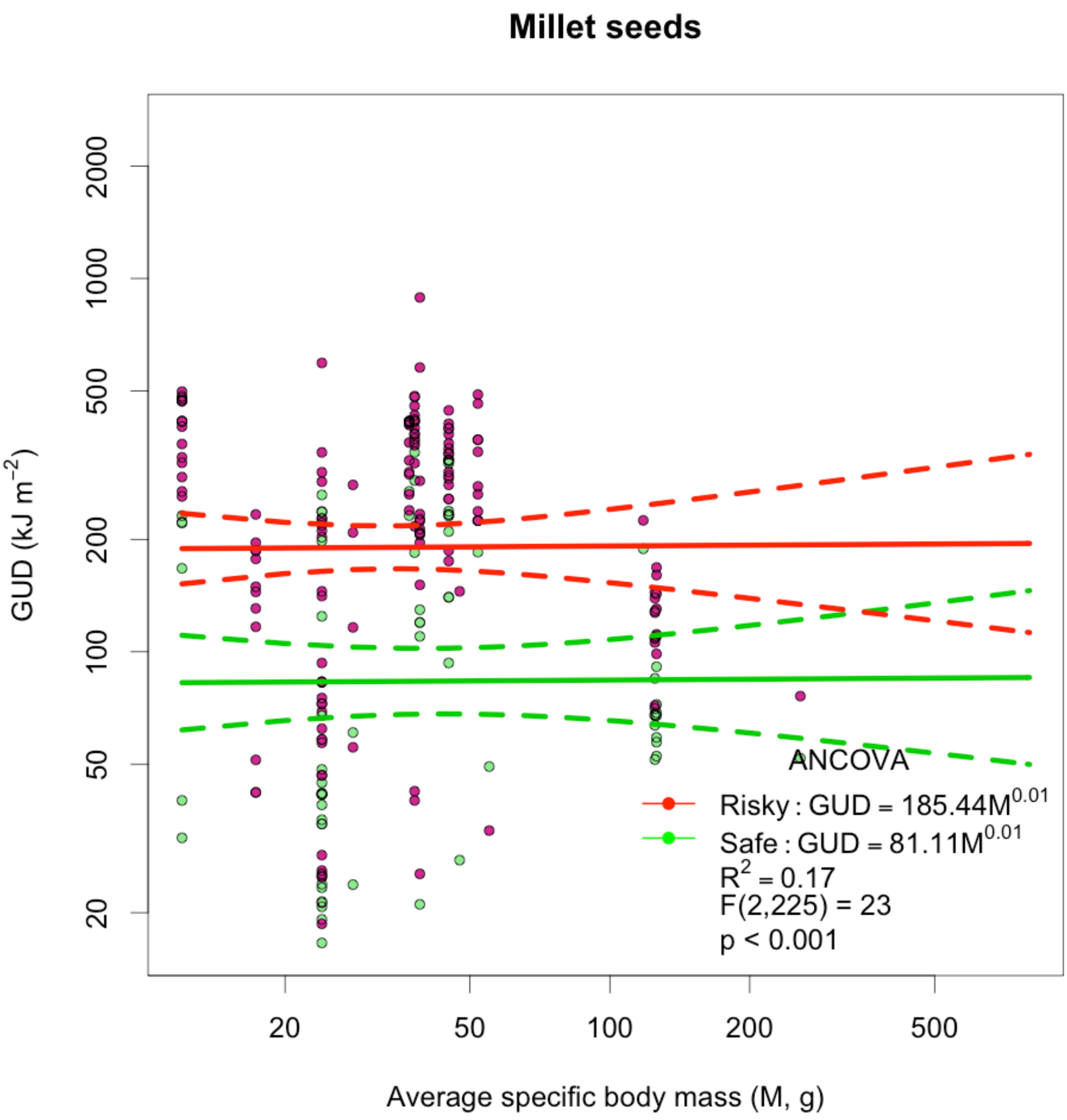

Figure S8: Average species body mass (M, g) scaling of Giving Up Densities (GUD, %) in risky (red) and safe (green) patches. The full lines represent the average scaling trend. The dashed lines represent the 95% Confidence Intervals around the average. The best selected model allows variations in intercept only across risk levels (Table 2).

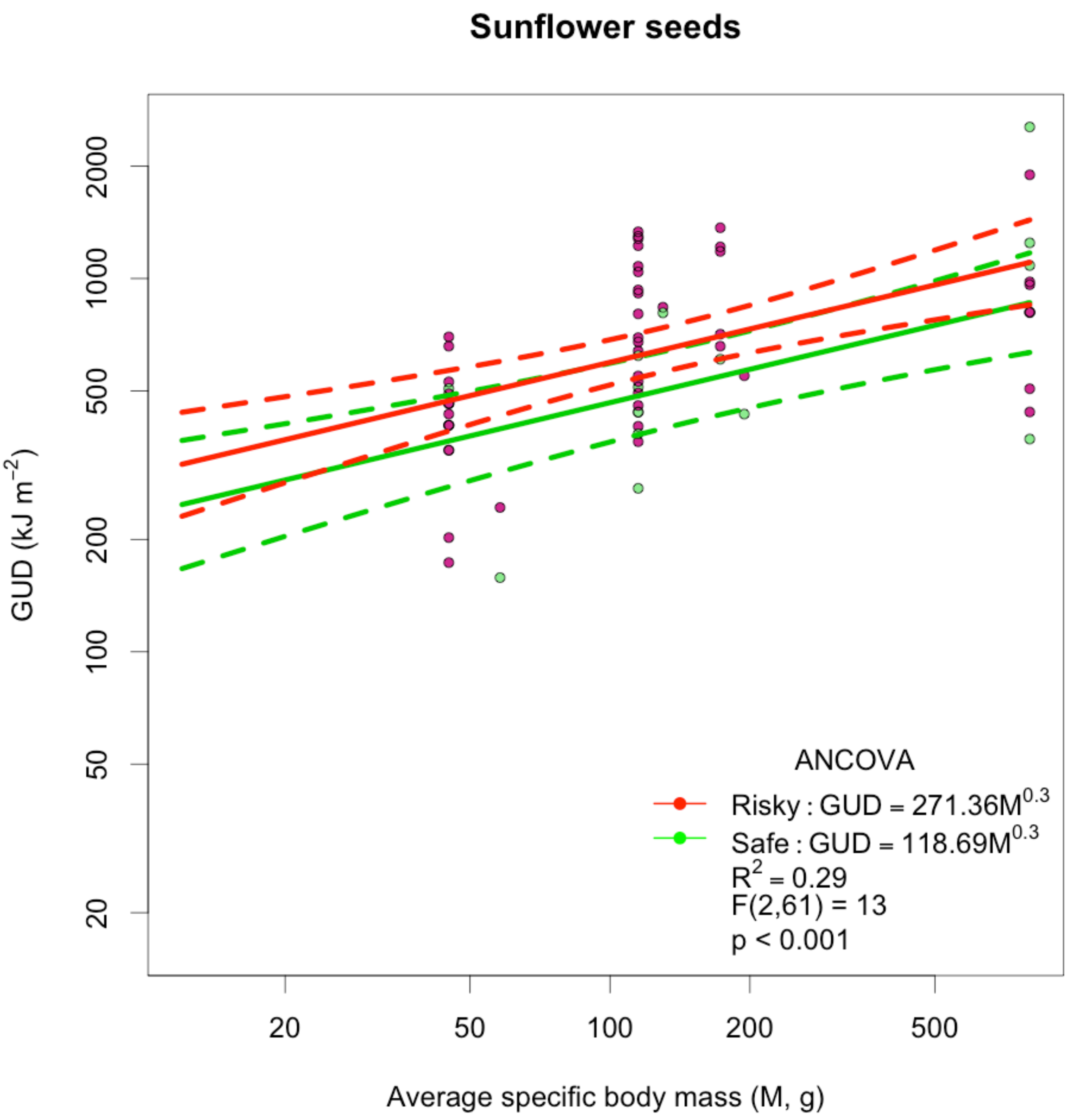

Figure S9: Average species body mass (M, g) scaling of Giving Up Densities (GUD, %) in risky (red) and safe (green) patches. The full lines represent the average scaling trend. The dashed lines represent the 95% Confidence Intervals around the average. The best selected model allows variations in intercept only across risk levels (Table 2).

Table S3: Comparison of linear models based on average species size (g), risk associated with foraging (safe vs. risky patches) and Giving Up Density of resources (GUD, kJ m-2) separately for treatments using millet seeds and treatments using sunflower seeds as food resourc. The continuous variables were natural log transformed. The full-interaction model and the cumulative model (best fit, bold) are shown.

	**GUD Millet (kJ m^{-2})†**			**GUD Sunflower (kJ m^{-2})‡**		
Predictors	*Estimates*	*95% CI*	*p*	*Estimates*	*95% CI*	*p*
Intercept	4.40	3.74 – 5.05	<0.001	4.78	4.13 – 5.43	<0.001
Size	0.01	-0.16 – 0.17	0.930	0.30	0.18 – 0.42	<0.001
Risk	0.83	0.58 – 1.07	<0.001	0.25	-0.02 – 0.52	0.074

Note: Total observations for Millet are 228 and for Sunflower are 64.

† R^2 / adjusted R^2 = 0.168 / 0.160
‡ R^2 / adjusted R^2 = 0.293 / 0.270

Table S4: ANOVA comparison among linear mixed models of the GUD~Size allometric relationship including Habitat as random effect and allowing random variations in intercept only or in both intercept and slope. The best model including habitat variations does not predict any variation for the effect of risk, while it allows variation in intercept and slope at the Habitat level (bold).

mod01<-lm(log(yy)~log(Size_g))

mod02<-lm(log(yy)~Risk)

mod03<-lm(log(yy)~log(Size_g)+Risk)

mod04<-lm(log(yy)~log(Size_g)*Risk)

modjj1<-lmer(log(yy)~log(Size_g)+Risk+(1|Habitat))

modjj2<-lmer(log(yy)~log(Size_g)+Risk+(log(Size_g)|Habitat))

modjj3<-lmer(log(yy)~log(Size_g)+Risk+(Risk|Habitat))

modjj4<-lmer(log(yy)~log(Size_g)+Risk+(log(Size_g)+Risk|Habitat))

modjj5<-lmer(log(yy)~log(Size_g)*Risk+(1|Habitat))

modjj6<-lmer(log(yy)~log(Size_g)*Risk+(log(Size_g)|Habitat))

modjj7<-lmer(log(yy)~log(Size_g)*Risk+(Risk|Habitat))

modjj8<-lmer(log(yy)~log(Size_g)*Risk+(log(Size_g)*Risk|Habitat))

	Df	AIC	BIC	logLik	deviance	Chisq	Chi Df	p
mod01	3	812	823	-403	806	NA	NA	NA
mod02	3	822	833	-408	816	0	0	1
mod03	4	767	781	-379	759	57	1	0
modjj1	5	747	765	-368	737	22	1	0
mod04	5	767	785	-379	757	0	0	1
modjj5	6	746	768	-367	734	23	1	0
**modjj2**	**7**	**745**	**771**	**-366**	**731**	**3**	**1**	**0,09343**
modjj3	7	749	775	-368	735	0	0	1
modjj6	8	745	774	-364	728	7	1	0,00695
modjj7	8	750	779	-367	734	0	0	1
modjj4	10	750	787	-365	730	4	2	0,17008
modjj8	15	757	812	-363	727	3	5	0,66482

Symbology:

lm(y~x_1) linear model
lm(y~x_1+x_2) linear model, cumulative interactions among explanatory variables (variation in intercept)
lm(y~x_1*x_2) linear model, multiplicative interactions among explanatory variables (variation in intercept and slope)
lmer(y~x_1+(1|r_1)) linear mixed model, variations in intercept only among random variables
lmer(y~x_1+(x_1|r_1)) linear mixed model, variations in intercept and slope among random variables

Table S5: ANOVA comparison among linear mixed models of the GUD~Size allometric relationship including Suborder as random effect and allowing random variations in intercept only or in both intercept and slope. The best model including habitat variations does not predict any variation for the effect of risk, while it allows variation in intercept and slope at the Suborder level (bold).

mod01=lm(log(yy)~log(Size_g)

mod02=lm(log(yy)~Risk)

mod03=lm(log(yy)~log(Size_g)+Risk)

mod04=lm(log(yy)~log(Size_g)*Risk)

modjs1=lmer(log(yy)~log(Size_g)+Risk+(1|Suborder))

modjs2=lmer(log(yy)~log(Size_g)+Risk+(log(Size_g)|Suborder))

modjs3=lmer(log(yy)~log(Size_g)+Risk+(Risk|Suborder))

modjs4=lmer(log(yy)~log(Size_g)+Risk+(log(Size_g)+Risk|Suborder))

modjs5=lmer(log(yy)~log(Size_g)*Risk+(1|Suborder))

modjs6=lmer(log(yy)~log(Size_g)*Risk+(log(Size_g)|Suborder))

modjs7=lmer(log(yy)~log(Size_g)*Risk+(Risk|Suborder))

modjs8=lmer(log(yy)~log(Size_g)*Risk+(log(Size_g)*Risk|Suborder))

	Df	AIC	BIC	logLik	deviance	Chisq	Chi Df	p
mod01	3	812	823	-403	806	NA	NA	NA
mod02	3	822	833	-408	816	0	0	1
mod03	4	767	781	-379	759	57	1	0
modjs1	5	712	731	-351	702	56	1	0
mod04	5	767	785	-379	757	0	0	1
modjs5	6	712	734	-350	700	57	1	0
**modjs2**	**7**	**680**	**705**	**-333**	**666**	**34**	**1**	**0**
modjs3	7	716	742	-351	702	0	0	1
modjs6	8	680	709	-332	664	38	1	0
modjs7	8	713	742	-348	697	0	0	1
modjs4	10	685	722	-333	665	31	2	0
modjs8	15	689	745	-330	659	6	5	0,31655

Symbology:

lm($y \sim x_1$) linear model

lm($y \sim x_1+x_2$) linear model, cumulative interactions among explanatory variables (variation in intercept)

lm($y \sim x_1*x_2$) linear model, multiplicative interactions among explanatory variables (variation in intercept and slope)

lmer($y \sim x_1+(1|r_1)$) linear mixed model, variations in intercept only among random variables

lmer($y \sim x_1+(x_1|r_1)$) linear mixed model, variations in intercept and slope among random variables
