# Supplementary material for: Size dependency of patch departure behavior: evidence from granivorous rodents
Source: Ecology. 2019 Jul 24;100(10):e02800. doi: 10.1002/ecy.2800 (PMC6852180; doi:10.1002/ecy.2800)
Supplement: Supplementary file 3 [file ECY-100-na-s003.pdf]

Cozzoli, F., Gjoni, V., Basset A., 2019. Size dependency of patch departure behavior: evidence from granivorous rodents. *Ecology*.

Data S1

Giving up densities of granivorous rodents

Authors

Francesco Cozzoli
Department of Biological and Environmental Sciences and Technologies
University of Salento
S.P. Lecce-Monteroni
73100 Lecce
Italy
Email: francesco.cozzoli@unisalento.it

Vojsava Gjoni
Department of Biological and Environmental Sciences and Technologies
University of Salento
S.P. Lecce-Monteroni
73100 Lecce
Italy

Alberto Basset
Department of Biological and Environmental Sciences and Technologies
University of Salento
S.P. Lecce-Monteroni
73100 Lecce
Italy

File list (file found within DataS1.zip)

`DATAS1.csv`
`DATAS1_REFERENCE_LIST.csv`

Description

`DATAS1.csv`: Giving Up Densities of granivorous rodents. We included in our meta-analysis 543 Giving Up Density (GUD) measures reported from 57 papers (`Data S1`

Reference list). The metadaset includes data on 40 granivorous rodents species belonging to 3 taxonomic suborders, 3 habitat typologies and 8 combinations of patch risk conditions patch risk conditions (Exposure, Illumination, Predation, 0 means that the risk factor has not been explicitly included in the experimental design). The provided amount and the typology of resource have been reported. GUD values have been reported both in g and kJ m^{-2} (Data S1 Conversion Table). Average species sizes (g) were obtained from the ADW - http://animaldiversity.org/accounts/Rodentia/ and website and AnAge - http://genomics.senescence.info. The size of the resource tray (surface, cm^2; volume, L; depth, cm) are also reported. Only 292 observations were used in the main analysis, while the others were excluded because they were not respecting one or more of the following conditions: i) foragers provided with resources other than the most commonly used (millet or sunflower seeds); ii) seeds dispersed in substrata other than the most commonly used (loose sand); iii) seeds dispersed in a disproportionately low volume of sand (<2 L); iv) size of the food trays not clearly reported v) repeated foraging episodes by multiple individuals of the same species not allowed; vi) foragers were provided with a disproportionately high amount of resources compared to other studies (>30 g).

Energetic contents of provided resources (kJ g-1, source http://foodnutritiontable.com):

Millet seeds	14 kJ g^{-1}
Peanut seeds	26 kJ g^{-1}
Sunflower seeds	26 kJ g^{-1}
Oat seeds	16 kJ g^{-1}
Wheat seeds	13 kJ g^{-1}
Garbanzo seeds	14 kJ g^{-1}
Alfalfa seeds	2 kJ g^{-1}
Line seeds	20 kJ g^{-1}
